# Evaluating the efficacy of purchased antisense oligonucleotides to reduce mouse and human tau *in vivo*

**DOI:** 10.3389/fnmol.2023.1320182

**Published:** 2023-12-18

**Authors:** Pranav Vemula, Kathleen M. Schoch, Timothy M. Miller

**Affiliations:** Department of Neurology, Hope Center for Neurological Disorders, Washington University in St. Louis School of Medicine, St. Louis, MO, United States

**Keywords:** antisense oligonucleotides, Alzheimer’s disease, tauopathies, tau protein, human tau mouse model

## Abstract

Many preclinical and clinical studies support the use of antisense oligonucleotides (ASOs) as effective therapeutic strategies. However, acquiring ASOs for research purposes may be limited by partnerships with the pharmaceutical companies. Our lab previously developed an effective ASO strategy to lower human tau and reverse pathology in aged tauopathy model mice. Testing the efficacy of purchased tau lowering ASOs would provide support for these reagents as broad research tools. Purchased mouse and human tau lowering ASOs were infused or injected intracerebroventricularly into wildtype and tau transgenic mice. Following treatment, brain tissue evaluated for ASO distribution and levels of tau mRNA, protein, and phosphorylated tau. We show that purchased ASOs enter cell types of the brain and effectively decrease mouse or human tau mRNA and protein levels. Human tau lowering ASO treatment in PS19 mice decreased phosphorylated tau and gliosis relative to saline-treated PS19 mice, consistent with our previous study using a non-commercial tau lowering ASO. The results of this study demonstrate the efficacy of purchased tau targeting ASOs *in vivo* to support their broad use by researchers.

## Introduction

A key shared feature of neurodegenerative diseases is the aberrant expression or accumulation of dysfunctional proteins. Tauopathies, including Alzheimer’s disease (AD), are the most common neurodegenerative diseases characterized by the abnormal aggregation of the microtubule associated protein tau (MAPT) in the brain (Götz et al., 2019). Various strategies targeting mechanisms to reduce tau pathology have been proposed and several tau-directed therapies are being investigated in clinical trials (Congdon and Sigurdsson, 2018; Jadhav et al., 2019; Guo et al., 2022). Among these interventions, gene targeting strategies, like antisense oligonucleotides (ASOs) that target mRNA to alter the protein expression, could be a viable approach, enabling studies that both investigate disease mechanisms and test therapeutic efficacy.

ASOs are single-stranded DNA-like sequences composed of a phosphate backbone and sugar rings, 8–50 base pairs in length, that bind complementarily via Watson-Crick-Franklin base pairing to target RNA (Schoch and Miller, 2017). Unmodified ASOs are prone to degradation; therefore, chemical modifications made to the phosphate backbone and sugar rings decrease or prevent degradation by nucleases and enhance target affinity (DeVos and Miller, 2013a; Schoch and Miller, 2017). ASOs exert their effect via various mechanisms, for example, degrading target mRNA by recruiting RNaseH, splicing modification by including or excluding exons, or inhibiting miRNA binding to the target mRNA., The strategy of RNaseH-mediated mRNA degradation has been used to lower the production of disease-causing genes/proteins in several diseases including amyotrophic lateral sclerosis (Smith et al., 2006; Jiang et al., 2016; Boros et al., 2022), Huntington’s disease (Kordasiewicz Holly et al., 2012; Lane et al., 2018; Kingwell, 2021), and tauopathies (DeVos et al., 2017).

In preclinical testing, human tau-targeted ASOs reduced phosphorylated tau deposition, rescued hippocampal loss, and increased survival in a tauopathy mouse model (DeVos et al., 2017). Intrathecal administrations of IONIS-MAPT_Rx_ (a second-generation tau lowering ASO) in non-human primates resulted in a mean *MAPT* mRNA reduction of 77% in the frontal cortex and 74% in the hippocampus without side effects (DeVos et al., 2017). Currently, human tau lowering ASOs are continuing through clinical trial testing with a recent successful phase 1b study having met its primary objective of safety and tolerability. In this phase 1b trial, a dose-dependent reduction in the concentration of total tau in the CSF was also observed in the patients receiving tau lowering ASO relative to the placebo group (Mummery et al., 2021). Together, these studies support direct targeting of tau mRNA as a promising therapeutic strategy for tauopathies, including AD.

Given their experimental and clinical utility, ASOs are considered powerful research tools to inform upon disease mechanisms prompting many researchers to use ASOs in their research. However, the design and synthesis of ASOs often requires partnerships with pharmaceutical industries which may be not feasible for every researcher. Several companies can also create ASO reagents, but these must be tested by the end-user. Here, we obtained ASOs from a commercial vendor designed to lower mouse or human tau mRNA and tested their efficacy in various mouse models. Both mouse tau lowering and human tau lowering ASOs significantly reduced their target mRNA and protein. When tested in the PS19 mouse model of tauopathy, the human tau lowering ASO was also effective at ameliorating phosphorylated tau pathology and gliosis, consistent with previous studies. Purchased tau-targeted ASOs effectively reduce mouse or human tau *in vivo*, establishing these ASO reagents as tools for tau-directed studies.

## Materials and methods

### Mice

Male C57BL/6 mice were purchased from Jackson Laboratories (stock number 000664) and used for ASO treatment studies at 4 months of age. hTau transgenic mice (Andorfer et al., 2003) and PS19 transgenic mice (Yoshiyama et al., 2007) were bred in-house and identified for transgene expression as previously described (Schoch et al., 2016; DeVos et al., 2017). For ASO treatment studies, we used male and female hTau mice at approximately 9 months of age and male and female PS19 mice and non-transgenic littermates at approximately 6 months of age. Animals were maintained on a 12-h light/dark cycle and provided *ad libitum* access to food and water. All husbandry and experimental procedures were approved by the Institutional Animal Care and Use Committee at Washington University in St Louis School of Medicine.

### Antisense oligonucleotides

Antisense oligonucleotides (ASOs) were purchased from Integrated DNA Technologies (IDT, Coralville, Iowa, USA) designed with 2′-O-methoxyethyl (2′-MOE) sugar ring and phosphorothioate (PS) backbone modifications. The 2′-MOE modification is well-tolerated, enhances target affinity, and increases resistance to degradation by nucleases while the PS modification enhances stability and cellular uptake (Schoch and Miller, 2017). Tau lowering ASOs were designed using a “gapmer” strategy, which consists of 2′-MOE modifications flanking the central region of unmodified nucleotides. Control ASOs with same modifications but without a specific target were included in select experiments. Synthesized ASOs were subjected to standard desalting procedure to remove small organic contaminants and high performance liquid chromatography (HPLC) purification to separate full-length oligonucleotides from truncated species based on hydrophobicity or charge difference. Sodium salt exchange was then applied on these ASOs to remove triethylammonium acetate residue from HPLC purification. ASO names and sequences are listed in the Supplementary Table 1. Control and tau-targeting sequences were identical in both sequence and chemistry to previously published studies in C57BL/6 (DeVos et al., 2013), hTau (Self et al., 2018), and PS19 mice (DeVos et al., 2017).

### Surgical procedures, euthanasia, and tissue dissection

C57BL/6, hTau, and PS19 mice were anesthetized with isoflurane via continuous inhalation and placed into a stereotaxic frame during surgical procedures. For ASO infusion into the lateral ventricle of C57BL/6 and PS19 mice, 28-day ALZET osmotic pumps, which deliver ASOs at a rate of 6 μl/day (equivalent to 30 μg/day) (Durect, Model 2004) were implanted as previously described (DeVos and Miller, 2013b). hTau mice received a single intracerebroventricular (ICV) bolus injection (300 μg) for a treatment duration of 1 month as previously described (DeVos and Miller, 2013b). For euthanasia and tissue collection, mice were anesthetized with isoflurane and perfused with ice-cold 1X phosphate buffered saline (PBS). Brains were collected rapidly, and the left hemisphere (contralateral to the catheter placement or ICV injection) was drop-fixed into ice-cold 4% paraformaldehyde for histological processing. The right hemisphere (ipsilateral to the catheter placement or ICV injection) was dissected into separate pieces for RNA and protein analyses, snap frozen in liquid nitrogen, and stored at −80^°^C until use.

### RNA isolation and quantitative RT-PCR analysis

Total RNA was extracted from right hemisphere brain lysates using a QIAGEN RNeasy kit (Catalogue #74104, QIAGEN, Hilden, Germany) per manufacturer’s instructions. Gene targets were amplified using Express One-Step Superscript qRT-PCR universal kit (Catalogue #11781200, ThermoFisher Scientific) and measured on a QuantStudio 12K Flex Real-Time PCR system (ThermoFisher Scientific). Target mRNA expression levels were normalized to mouse *Gapdh* mRNA levels and calculated using the ΔΔCt method. Primer and probe sequences were as follows: mouse *Mapt*: forward 5′-GAACCACCAAAATCCGGAGA-3′, reverse 5′-CTCT TACTAGCTGATGGTGAC-3′, probe 5′-/56-FAM/CCAAGAAGG TGGCAGTGGTCC/3IABkFQ/-3′; human *Mapt*: forward 5′-AG AAGCAGGCATTGGAGAC-3′, reverse 5′-TCTTCGTTTTACC ATCAGCC-3′, probe 5′-/56-FAM/ACGGGACTGGAAGCGATGA CAAAA/3IABkFQ/-3′; *Gapdh*: forward 5′-TGCCCCCATG TTGTGATG-3′, reverse 5′-TGTGGTCATGAGCCCTTCC-3′, probe 5′-/56-FAM/AATGCATCCTGCACCACCAACTGCTT/3IA BkFQ/-3′.

### Protein homogenization and enzyme-linked immunosorbent assay (ELISA)

Total protein was extracted from tissue sections from the right hemisphere of mouse brain using RAB buffer (100 mM MES, 1 mM EDTA, 0.5 mM MgSO_4_, 750 mM NaCl, 20 mM NaF, 1 mM Na_3_VO_4_) containing phosphatase and protease inhibitors and homogenized with a handheld tissue homogenizer. Bicinchoninic Acid (BCA) assay was used to quantify extracted protein.

For quantification of human and mouse tau protein, sandwich ELISA of tau-5 coating antibody (20 μg/ml; Millipore Cat# 577801-100UG RRID:AB_212534) with either biotin-conjugated HT-7 (for human tau, 0.3 μg/ml; ThermoFisher Scientific Cat# MN1000B RRID:AB_223453) or BT-2 (for mouse tau, 0.3 μg/ml; ThermoFisher Scientific Cat# MN1010B RRID:AB_10974155) antibodies was performed. Initially, 96-half-well plates were coated and incubated overnight with tau-5 antibody diluted in carbonate coating buffer (0.2M NaHCO_3_/Na_2_CO_3_). The plates were then blocked with 4% bovine serum albumin (BSA)/PBS at 37^°^C. Standards of recombinant human tau (2N4R; rPeptide, Bogart, GA) or mouse tau (mTau40, 432aa, produced in the laboratory of Eva-Maria Mandelkow) and samples were diluted in sample buffer (0.25% BSA, 300 mM Tris, and 1X protease inhibitor cocktail in PBS), loaded on plates, and incubated overnight at 4^°^C. HT7 and BT-2 capture antibodies followed by streptavidin-poly-HRP-40 conjugate (1:4000; Fitzgerald, Acton, MA) were applied and detected by the addition of 3,3′,5,5′-tetramethylbenzidine liquid substrate, Super Slow (T5569, Sigma) reagent. The plates were read using a BioTek microplate reader (Biotek Epoch, Winooski, VT) at 650nM.

### Immunohistochemistry

The left hemisphere of mouse brains remained in 4% paraformaldehyde for 24 h, was transferred to a 30% sucrose solution for cryoprotection, and frozen in cold (−20 to −35°C) 2-methylbutane. Frozen brains were sectioned into 40 μm coronal sections using a microtome and stored free-floating in a cryoprotectant solution (30% ethylene glycol, 30% glycerol, 10% 0.2 M phosphate buffer, in water) at −20°C until use.

For immunofluorescence, three sections per brain (approximate bregma level range −1.75 to −3.00 mm) were selected from saline- and ASO-treated PS19 mice and non-transgenic littermates. All immunofluorescence procedures were performed at room temperature except primary antibody incubation. Sections were incubated in a Tris-buffered saline (TBS) −0.1% Triton X-100 blocking solution containing 2% normal donkey serum, 4% BSA, 0.1% hydrogen peroxide, and 100mM glycine and then incubated in primary antibody at 4^°^C overnight (Pan ASO 1:1000, gift from Ionis Pharmaceuticals, Carlsbad, CA, USA; NeuN 1:500, 266004 Synaptic Systems, Goettingen, Germany; GFAP 1:1000, AB5541 Millipore-Sigma; Iba1 1:500 ab5076 Abcam, Cambridge, United Kingdom). Sections were incubated in blocking solution prior to incubation in secondary antibodies (all at 1:1000; for ASO: anti-rabbit Alexa Fluor Plus 647, A32795 ThermoFisher Scientific; for NeuN: anti-guinea pig Alexa Fluor 555, A21435 Invitrogen or anti-chicken Alexa Fluor 594, 703-585-155 Jackson Immuno Research Labs, West Grove, PA, USA; for GFAP: anti-chicken Alexa Fluor 488, 703-545-155 Jackson Immuno Research Labs; for Iba1: anti-goat Alexa Fluor 488, 705-545-147 Jackson Immuno Research Labs). Sections were washed, incubated in DAPI (1:1000), and mounted on slides. Slides were covered with coverslips using Fluoromount-G mounting media (SouthernBiotech, Birmingham, AL, USA) and sealed with nail polish. Images were collected from the CA3 region of the hippocampus at bregma −1.85 mm using a Zeiss LSM 880 microscope (Zeiss, Oberkochen, Germany) with a 40 × 1.3NA oil objective and the Airyscan super-resolution detector with tile scanning. Following initial image acquisition, all images were Airyscan Processed using Zeiss Zen Blue 3.6. Overview images had an XY resolution of 70nm per pixel and are presented as a maximal intensity projections of a 1.2μm z-stack. For cell-specific imaging, a 10.2μm z-stack was collected with a resolution of 50nm in XY and 300nm in Z. Maximal intensity projections are shown for the XY plane of each identified cell to show clear cellular morphology and 1.8μm slices are shown for the XZ and YZ orthogonal projections to identify ASO that is localized within the specific cell types.

For immunohistochemistry of phosphorylated tau, six sections per brain (approximate bregma level range −1.65 to −2.98 mm) were selected from non-transgenic and saline- or ASO-treated PS19 mouse tissue. All immunohistochemical procedures were performed at the room temperature except primary antibody incubation. Sections were incubated in 0.3% hydrogen peroxide prior to blocking in 3% non-fat dry milk in PBS-0.25% Triton X-100 solution. Sections were then incubated in primary antibody at 4^°^C overnight (biotinylated AT8, 1:500, MN1020B, ThermoFisher Scientific). The biotin signal was amplified using an ABC Elite kit (1:400; PK-6100, Vector Laboratories, Burlingame, CA, USA) and detected by reaction with 3, 3‘-diaminobenzidine (DAB) solution (SK-4100, Vector Laboratories). Sections were then washed and mounted on slides. Mounted sections were dehydrated through an ethanol gradient followed by incubation in xylene. Slides were covered with coverslips using mounting media (Cytoseal 60, #8310-4, ThermoFisher Scientific) and imaged at 20x on a Hamamatsu NanoZoomer HT whole slide imager (Hamamatsu Photonics, Japan).

### Immunohistochemical image analysis

To quantify phosphorylated tau, section images from AT8 immunohistochemistry were exported at 2.5x (cortex) or 5x (hippocampus) magnification using the NDP view (Hamamatsu) program and analyzed in ImageJ as previously described (Schoch et al., 2016). In brief, cortical and hippocampal regions across six sections were outlined as ROIs and a uniform, global threshold used to identify AT8 + reactivity within each region. The amount of AT8 + reactivity was recorded as a percentage of the total ROI area.

To quantify gliosis, images from saline- and ASO-treated PS19 and non-transgenic littermate controls stained for Iba1 (microglia) and GFAP (astrocytes) were exported using ZEN 3.4 software (ZEISS) and analyzed in ImageJ. Hippocampal regions across three sections were outlined as ROIs and a uniform, global threshold used to identify Iba1 + or GFAP + reactivity within each region. The amount of reactivity was recorded as a percentage of the total ROI area.

### Statistical analysis

Data were represented in mean ± SEM and analyzed with GraphPad Prism (version 9). mRNA levels and protein expression were analyzed using unpaired *t*-test (for two groups) or ordinary one-way ANOVA with Tukey’s *post-hoc* test (for three or more groups). Analysis of immunohistochemical data (i.e., phosphorylated tau and gliosis) was done using one-way ANOVA with Tukey’s *post-hoc* test. A p value of < 0.05 was deemed significant.

## Results

### Purchased ASOs are taken up by neurons and glia

**FIGURE 1:**
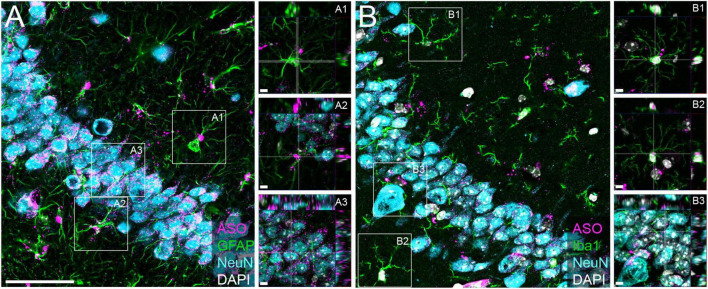
Purchased ASOs distribute to neurons and glia within the brain. Immunofluorescent images within the CA3 region of the hippocampus from mice treated with purchased ASOs showing ASO distribution within neurons (NeuN), astrocytes (GFAP), and microglia (Iba1). **(A)** Representative image of ASO, GFAP, and NeuN and **(B)** ASO, Iba1, and NeuN immunoreactivity, with orthogonal projection image insets to show cell-specific localization. Scale bars = 50 μm, insets 5 μm.

We have previously demonstrated that tau-targeted ASOs reliably distribute throughout the brain and can be taken up by neurons and glia in the central nervous system (CNS) (DeVos et al., 2013, 2017). To test the ability of a purchased ASO to distribute and localize to cell types within the CNS, we performed immunofluorescence to visualize localization of the ASO in neurons, microglia, and astrocytes. We noted co-localization of the ASO with NeuN (Figures 1A3, B3), Iba1 (Figures 1B1, B2), and GFAP (Figures 1A1, A2), confirming neuronal and glial uptake of ASO.

### Mouse tau knockdown in C57BL/6 mice reduces tau expression

**FIGURE 2:**
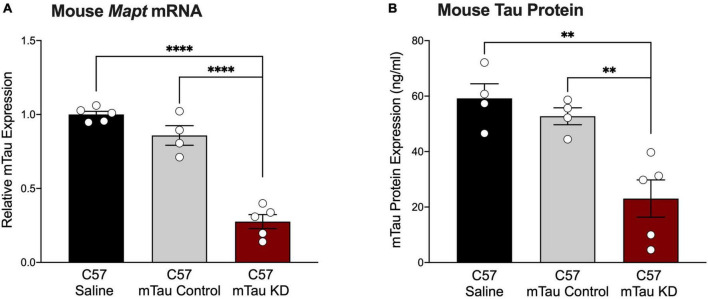
Purchased mouse tau-targeting ASO reagents reduce mouse tau mRNA and protein in C57BL/6 mice. **(A)** Mouse tau mRNA expression was significantly decreased following administration of purchased mouse tau knockdown (mTau KD) ASO in C57BL/6 (C57) mice relative to saline and control ASO treated mice (*n* = 4–5/group). Mouse tau mRNA levels were normalized to *Gapdh* and shown relative to saline treated mice. One-way ANOVA, *F* = 74.68, *P* < 0.0001; Tukey’s multiple comparisons test. **(B)** Mouse tau protein expression was significantly decreased in mTau KD ASO-treated mice relative to saline or control ASO-treated mice (n = 4–5/group). One-way ANOVA, *F* = 12.69, *P* < 0.01; Tukey’s multiple comparisons test. Data are presented as mean ± SEM. **P < 0.01, *⁣*⁣**P < 0.0001.

Although mouse tau does not form pathological tau aggregates in wildtype mice, mouse tau may be used as a target to inform upon the physiological function of tau within the CNS. To test the effect of a purchased mouse tau-targeted ASO, we administered mouse tau knockdown (mTau KD) ASO via intraventricular osmotic pump to C57BL/6 mice and measured mTau mRNA expression 2 months after pump implantation. C57BL/6 mice treated with mTau KD ASO showed significant reduction in the levels of mTau mRNA expression levels with 73% and 68% decrease (Figure 2A) relative to saline or control ASO treated mice, respectively. No significant difference in mTau mRNA expression was observed between saline and control ASO treated mice (*P* = 0.1265). We also measured changes in the protein expression of mTau protein after ASO administration by ELISA. We found reduction in the levels of mTau protein in mice treated with mTau KD ASO with 61% and 56% decrease (Figure 2B) compared to saline or control ASO treated mice, respectively.

### Human tau knockdown in hTau mice reduces human tau mRNA and protein expression

**FIGURE 3:**
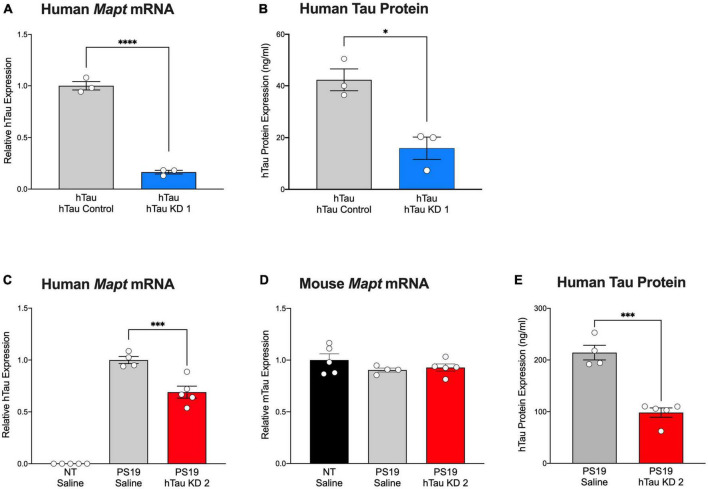
Purchased human tau-targeting ASO reagents reduce human tau mRNA and protein in hTau and PS19 mice. **(A)** Human tau mRNA and **(B)** human tau protein expression were significantly decreased following purchased human tau knockdown (hTau KD 1) ASO administration in hTau mice relative to control ASO treated hTau mice (*n* = 3/group). Human tau mRNA levels were normalized to *Gapdh* and shown relative to control ASO. For Panel **(A)**: Two-tailed *t*-test, *P* < 0.0001; Panel **(B)**: Two-tailed *t*-test, *P* < 0.05. **(C)** Human tau mRNA expression was significantly decreased following treatment with purchased human tau knockdown (hTau KD 2) ASO in PS19 mice relative to saline treatment (*n* = 4–5/group). Human tau mRNA expression was undetected in non-transgenic (NT) littermates treated with saline (*n* = 5). One-way ANOVA, F = 164.9, *P* < 0.0001; Tukey’s multiple comparisons test. **(D)** No difference in mouse tau mRNA levels was observed among treated mice. mRNA levels were normalized to *Gapdh*and expressed relative to saline-treated mice. One-way ANOVA, *F* = 1.258, *P* = 0.3221. **(E)** Human tau protein expression was significantly reduced in hTau KD 2 ASO-treated PS19 mice relative to saline-treated PS19 mice. Two-tailed *t*-test, *P* < 0.001. Data are presented as mean ± SEM. *P < 0.05, ***P < 0.001, ****P < 0.0001.

Human tau mice (hTau mice) express all six isoforms of human tau under control of the human tau promoter in the absence of endogenous mouse tau (Andorfer et al., 2003), offering a model that closely mirrors human tau expression. Our previous study in hTau mice treated with hTau KD ASO showed significant reduction in human tau mRNA and protein levels (Self et al., 2018). To test the effect of the purchased hTau KD ASO, we administered hTau KD ASO 1 via single ICV injection (similar treatment paradigm to our previous study (Self et al., 2018)) to hTau mice and measured hTau mRNA and protein levels. hTau KD ASO 1 treatment afforded a significant reduction (84% decrease) in the levels of human tau mRNA expression levels relative to control ASO (Figure 3A). We also measured human tau protein expression using ELISA after hTau KD ASO 1 administration in hTau mice. ASO-mediated hTau knockdown in hTau mice showed a significant reduction (62% decreases) in human tau protein expression relative to control ASO treated hTau mice (Figure 3B).

### Human tau knockdown in PS19 mice reduces human tau mRNA and attenuates pathology and gliosis

Mutant tau expression in PS19 closely recapitulates the time course of tau phosphorylation and aggregation, neuroinflammation, and cell loss evident in human tauopathies (Yoshiyama et al., 2007). In our past studies, we demonstrated the ability of hTau-lowering ASOs to reverse tau pathology and extend survival in PS19 mice (DeVos et al., 2017). Using a purchased version of the ASO, we administered hTau KD ASO 2 via osmotic pump to PS19 mice and measured human tau mRNA and protein expression 2 months after ASO administration. Non-transgenic littermates treated with saline were included as treatment controls. hTau KD ASO 2 treated mice showed a significant reduction in the levels of human tau mRNA (31% decrease) relative to saline treated mice without a change in mouse tau mRNA expression, confirming the specificity of the human tau target (Figures 3C, D). We observed reduced human tau protein expression in PS19 mice treated with hTau KD ASO (54% decrease) relative to saline treated PS19 mice (Figure 3E).

**FIGURE 4:**
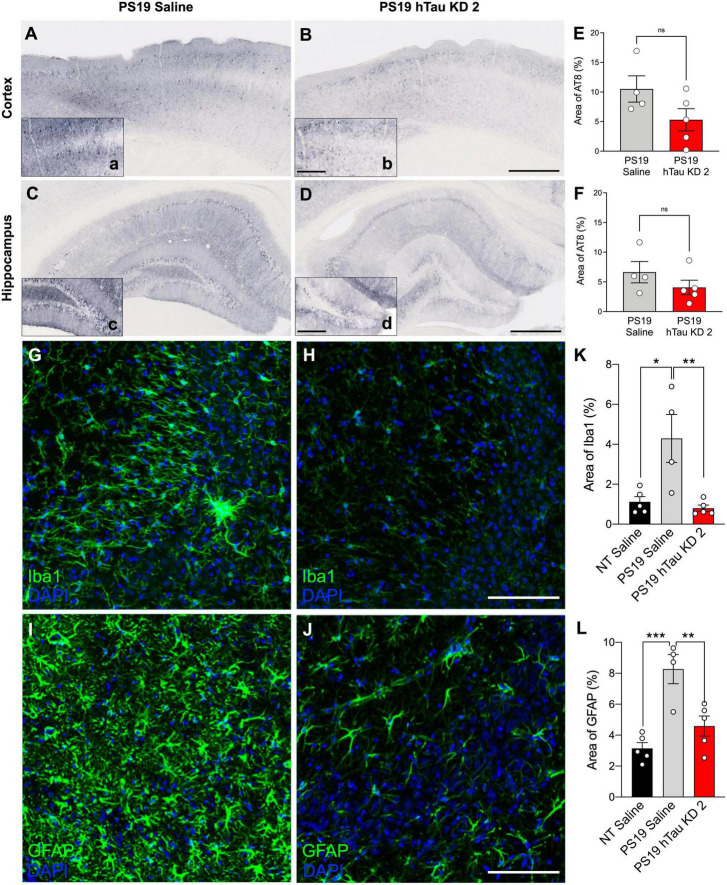
hTau KD ASO treatment in PS19 mice shows a reduced trend in phosphorylated tau accumulation and reduces gliosis. Representative images of phosphorylated tau (AT8) immunohistochemistry within the cortex and hippocampus from **(A–C)** saline-treated PS19 mice and **(B–D)** human tau knockdown (hTau KD 2) ASO-treated PS19 mice. Scale bar = 500 μm, insets 250 μm. Quantification of the percentage of AT8 immunoreactivity within the **(E)** cortex and **(F)** hippocampus of saline or hTau KD 2 treated PS19 mice (*n* = 4–5 mice/group). Panel **(E)**, Cortex: One-way ANOVA, *F* = 10.50, *P* = 0.0028, Tukey’s multiple comparison test, *P* = 0.1032; Panel **(F)**, Hippocampus: One-way ANOVA, *F* = 8.162, *P* = 0.0067, Tukey’s multiple comparison test, *P* = 0.3126. Representative images from the CA3 region of the hippocampus showing **(G,H)** Iba1 and **(I,J)** GFAP immunofluorescence for microglia and astrocytes, respectively, from PS19 mice treated with saline or human tau knockdown (hTau KD 2) ASO. Scale bars = 100 μm. Quantification of the percentage of **(K)** Iba1 and **(L)** GFAP immunoreactivity within the hippocampus of non-transgenic (NT) and saline or hTau KD treated PS19 mice (*n* = 4–5 mice/group). Panel **(K)**, Iba1: One-way ANOVA, *F* = 9.216, *P* < 0.01, Tukey’s multiple comparison test, *P* < 0.01; Panel **(L)**, GFAP, *F* = 15.20, *P* < 0.001; Tukey’s multiple comparison test, *P* < 0.01. Data are presented as mean ± SEM. *P < 0.05, **P < 0.01, ***P < 0.001, and ns, non-significant.

PS19 mice develop phosphorylated tau at 6 months of age, accompanied by significant microgliosis beginning at 3 months age and astrogliosis at 6 months age (Yoshiyama et al., 2007). To investigate pathological outcomes in PS19 mice after hTau lowering with a purchased ASO, we performed immunohistochemistry using the AT8 antibody to identify phosphorylated tau reactivity within the cortex and hippocampus (Figures 4A–D). PS19 mice treated with hTau KD ASO 2 exhibited reduced phosphorylated tau staining compared to saline treated PS19 mice in both the cortex (49% decrease) and hippocampus (39% decrease), although not statistically significant (Figures 4E, F). Next, to test the effect of hTau KD ASO 2 on microglial and astrocytic activation, we probed mouse brain sections with antibodies to Iba1 (Figures 4G, H) and GFAP (Figures 4I, J). PS19 mice treated with hTau KD ASO 2 showed significant reductions in Iba1 (81% decrease) and GFAP (45% decrease) relative to saline treated mice (Figures 4K, L).

## Discussion

We tested mouse and human tau lowering ASOs purchased from a commercial vendor in adult mice to support their use as research tools for tau-directed studies. Various studies demonstrated that ASOs show widespread distribution and activity in various brain regions and cell types (Jafar-Nejad et al., 2021; Mortberg et al., 2023). A recent study reported ASO distribution and activity across various CNS cell types and brain regions using single nucleus RNA-sequencing. They found that cell types such as neurons, astrocytes, and microglia show similar residual target mRNA levels (in the cortex) 3 weeks post ASO treatment citing similar efficiency of ASO uptake by these cell types. On the other hand, in thalamus and cerebellum, microglia and astrocytes show less residual target mRNA levels than in neurons perhaps indicating more efficient ASO uptake in glia than neurons (Mortberg et al., 2023). We identified that the ASOs can be taken up by neurons and glia within the CNS, suggesting widespread distribution and uptake consistent with prior studies. Mouse- and human tau-targeted ASOs were able to reduce mouse or human tau mRNA and protein levels when administered to wildtype or human tau-expressing mice (hTau and PS19 mice), respectively. When administered to PS19 mice, hTau KD ASO also reduced phosphorylated tau deposition and gliosis.

Advancements in ASO chemistry coupled with our understandings of RNA biology have led to an evolution of ASO design and application (Crooke et al., 2021). Although the ASO sequences and modifications used in our studies were previously determined, designing ASOs to target a gene of interest has become feasible with freely available computational algorithms to generate potential ASO candidates for subsequent testing. When selecting potential ASOs for screening, factors such as RNA secondary structure, GC content of the ASO, and binding energy (ΔG°37) are considered (Chan et al., 2006) to achieve a maximally effective ASO. Finally, chemical modifications to the PS backbone and sugar rings render ASOs with increased stability, protection from nucleases, increased binding ability, tolerability, and the ability to recruit RNase H (Sud et al., 2014; DeVos et al., 2017; Chakravarthy et al., 2020; Crooke et al., 2021). Several companies offer ASO synthesis and purification services, expanding the availability of these resources for research purposes.

The purchased ASOs used in the current study were synthesized and chemically modified using previously published *MAPT* targeting sequences and designs (DeVos et al., 2013, 2017; Self et al., 2018). Similarly designed ASOs are predicted to have widespread distribution and activity within various brain regions and cell types (Jafar-Nejad et al., 2021; Mortberg et al., 2023). Consistent with this, we demonstrated purchased ASOs localized within neurons, astrocytes, and microglia. All ASOs tested effectively decreased *MAPT*, suggesting purchased ASO reagents are feasible for use *in vivo*. Previous studies have used tau targeting ASOs with alternative modifications showing similar efficacy. Sud et al. (2014) employed morpholino ASOs to target *MAPT* in the human neuroblastoma cell lines, SH-SY5Y and IMR32, and in tau transgenic mice. These ASOs reduced *MAPT* mRNA up to 50% and protein up to 80% (Sud et al., 2014). Another study demonstrated the ability of 2′-*O*-Methyl (2′-*O*-Me) modified ASOs on a PS backbone to reduce *MAPT* mRNA (92% reduction) and tau protein (50% reduction after 48-h treatment) levels *in vitro* in SH-SY5Y cells (Chakravarthy et al., 2020). These studies underscore the capability of ASOs *in vitro* and *in vivo* as excellent reagents to lower tau levels.

Importantly, the tau lowering ASOs tested here can target mutant human tau, enabling studies on tau as a therapeutic target in tau-mediated diseases. DeVos et al. (2017) demonstrated that use of human tau-targeted ASOs could reduce human tau mRNA and protein, reverse tau pathology, preserve neuronal number, and increase survival in aged PS19 mice (DeVos et al., 2017). Consistent with this study, the purchased equivalent ASO reduced human tau mRNA and protein; however, the level of human tau mRNA knockdown and protein reduction were slightly lower than that achieved in the study by DeVos et al. (2017). Although the identical sequence and chemical modifications were used in this study, differences in the synthesis or purification process may produce varying efficacies, with the purchased ASOs being less effective. In addition, the differences in the effect size between the studies may be due to varying responses of the mice to the ASO treatment or differences in surgical experience between surgeons. Purchased human tau knockdown ASO also afforded reductions of phosphorylated tau in the cortex and hippocampus of PS19 mice relative to saline treatment, although these reductions did not achieve statistical significance. The discrepancy in the effect size of tau ASOs on pathological readouts is likely due to the difference in treatment duration [2 months in the present study as opposed to 3 months in DeVos et al. (2017)]. Also, we note the high degree of variability in AT8 reactivity within treatment groups, likely due to the inherent variability of the line and relatively small group sizes. In addition, both male and female PS19 mice were evaluated in DeVos et al. (2017), and sex-dependent differences in phosphorylated tau reactivity have been reported (Sun et al., 2020). Gliosis was significantly attenuated in hTau KD ASO-treated PS19 mice; neurodegeneration and survival measures would further confirm the efficacy of the ASOs in a pathological context. However, the mice in the present study were evaluated at 6 months age, prior to expected neuronal loss or premature death. Based on the ability of purchased ASOs to attenuate gliosis, we predict that these ASOs would be similarly effective at attenuating late-stage pathology or other deficits, supporting their use as a therapeutic agent for researchers studying tau-mediated disease mechanisms.

To date, ten ASO drugs have been approved by the United States Food and Drug Administration for a variety of diseases (Dhuri et al., 2020; Crooke et al., 2021). The use of ASOs in CNS disorders has continued to expand, and several ASOs targeting genes implicated in neurodegenerative diseases are currently in clinical trial or recently approved. For example, tofersen, a 2′-MOE ASO designed to target superoxide dismutase (*SOD1*) for amyotrophic lateral sclerosis (ALS), recently completed phase 3 clinical trial testing and received FDA approval in April 2023 (Miller et al., 2020, 2022; FDA, 2023). BIIB080, an ASO that targets human *MAPT* gene to prevent tau protein production for tauopathies including AD, has reported positive phase 1b results with evidence for tau reduction in the CSF of treated patients with mild AD (Mummery et al., 2021) and is in currently in Phase 2 trial in early Alzheimer’s Disease (NCT05399888). Despite these promising results, other ASOs were not effective in human clinical trials. For example, tominersen, a 2′-MOE ASO to target huntingtin for Huntington’s disease (ClinicalTrials.gov Identifier: NCT02519036; NCT03761849) (Tabrizi et al., 2019) showed no serious adverse effects in phase 2 trials, but phase 3 trials were stopped due to lack of efficacy. Similarly, further studies of a C9orf72 ASO for ALS were not pursued due to negative biomarker and clinical data from the initial clinical trial (Biogen, 2022). Regardless of these trial outcomes, preclinical testing of ASOs was vital to their successful transition into clinical application. ASO reagents that are accessible and effective may enable future therapeutic discoveries.

Alternative gene-targeting siRNA reagents or synthetic small molecules can serve as research tools or therapeutic drugs to achieve a similar effect to ASOs. siRNAs are synthetic 19–22 bp long double-stranded molecules that are complementary to target mRNA and can regulate mRNA expression (Watts and Corey, 2012). Like ASOs, siRNAs are advantageous because of their efficacy and unrestricted choice of targets. However, unlike ASOs, siRNAs often require a delivery agent such as liposomes or conjugation to cholesterol (de Fougerolles et al., 2007) for sufficient uptake. For small molecules, which are synthetic organic compounds that target proteins, a major advantage is their low molecular weight, which enables cellular penetration. However, their development involves a complicated process of targeted validation approaches, they often exhibit short half-lives requiring frequent administration, and they lack target specificity. While every drug modality has its advantages and limitations, target engagement is one of the most desirable drug properties, and ASOs, owing to their complementary base pairing, are highly specific. However, ASOs can elicit off-target toxicities and pro-inflammatory effects (Scoles and Pulst, 2018) and lack the ability to cross blood-brain barrier requiring direct administration into the CSF via lumbar puncture (DeVos and Miller, 2013a). Despite these limitations, the advantages of ASOs outweigh their alternatives and the ease of development and translatability make them excellent candidates as therapeutics (Schoch and Miller, 2017).

## Conclusion

Overall, the current study demonstrates the use and efficacy of purchased tau-targeting ASOs to show that easy access to ASOs through commercial vendors enables their broad use by researchers to address various research questions on disease mechanisms and potential therapeutics.

## Data availability statement

The original contributions presented in this study are included in this article/Supplementary material, further inquiries can be directed to the corresponding author.

## Ethics statement

The animal study was approved by the Institutional Animal Care and Use Committee (IACUC) at Washington University in St. Louis. The study was conducted in accordance with the local legislation and institutional requirements.

## Author contributions

PV: Writing–review and editing, Formal analysis, Investigation, Writing–original draft. KS: Writing–review and editing, Conceptualization. TM: Conceptualization, Writing–review and editing, Funding acquisition.
