# Supplementary material for: Evaluating the efficacy of purchased antisense oligonucleotides to reduce mouse and human tau in vivo
Source: Front Mol Neurosci. 2023 Dec 18;16:1320182. doi: 10.3389/fnmol.2023.1320182 (PMC10773814; doi:10.3389/fnmol.2023.1320182)
Supplement: Supplementary file 1 [file Table_1.DOCX]

Supplementary Material

**Supplementary Table:** Purchased control and tau-targeting ASOs used to evaluate tau lowering in mice. The species target and the mouse model in which the ASO was used are indicated. ASO modifications are indicated as follows: 2’-MOE nucleotides (red font), unmodified nucleotides (underlined). Nucleotides with PS backbone are indicated by asterisk.

| **ASO name** | **Target** | **Used in** | **ASO sequence (5’ to 3’)** |
| --- | --- | --- | --- |
| mTau^a^ Control | No target | C57BL/6 mice | C*C*T*T*C*C*C*T*G*A*A*G*G*T*T*C*C*T*C*C |
| hTau^b^ Control | No target | hTau mice | C*CTAT*A*G*G*A*C*T*A*T*C*C*AGG*A*A |
| mTau^a^ KD^c^ | Mouse tau | C57BL/6 mice | A*T*C*A*C*T*G*A*T*T*T*T*G*A*A*G*T*C*C*C |
| hTau KD 1 | Human tau | hTau mice | C*CGTT*T*T*C*T*T*A*C*C*ACC*C*T |
| hTau KD 2 | Human tau | PS19 mice | G*C*T*T*T*T*A*C*T*G*A*C*C*A*T*G*C*G*A*G |

^a^ mouse tau; ^b^ human tau; ^c^ knockdown.
